# Artificial intelligence based prediction model of in-hospital mortality among females with acute coronary syndrome: for the Jerusalem Platelets Thrombosis and Intervention in Cardiology (JUPITER-12) Study Group

**DOI:** 10.3389/fcvm.2024.1333252

**Published:** 2024-03-04

**Authors:** Ranel Loutati, Nimrod Perel, David Marmor, Tommer Maller, Louay Taha, Itshak Amsalem, Rafael Hitter, Manassra Mohammed, Nir Levi, Maayan Shrem, Motaz Amro, Mony Shuvy, Michael Glikson, Elad Asher

**Affiliations:** Jesselson Integrated Heart Center, Shaare Zedek Medical Center and Faculty of Medicine, Hebrew University of Jerusalem, Jerusalem, Israel

**Keywords:** artificial intelligence, machine learning, ACS, sex disparities, in-hospital mortality

## Abstract

**Introduction:**

Despite ongoing efforts to minimize sex bias in diagnosis and treatment of acute coronary syndrome (ACS), data still shows outcomes differences between sexes including higher risk of all-cause mortality rate among females. Hence, the aim of the current study was to examine sex differences in ACS in-hospital mortality, and to implement artificial intelligence (AI) models for prediction of in-hospital mortality among females with ACS.

**Methods:**

All ACS patients admitted to a tertiary care center intensive cardiac care unit (ICCU) between July 2019 and July 2023 were prospectively enrolled. The primary outcome was in-hospital mortality. Three prediction algorithms, including gradient boosting classifier (GBC) random forest classifier (RFC), and logistic regression (LR) were used to develop and validate prediction models for in-hospital mortality among females with ACS, using only available features at presentation.

**Results:**

A total of 2,346 ACS patients with a median age of 64 (IQR: 56–74) were included. Of them, 453 (19.3%) were female. Female patients had higher prevalence of NSTEMI (49.2% vs. 39.8%, *p* < 0.001), less urgent PCI (<2 h) rates (40.2% vs. 50.6%, *p* < 0.001), and more complications during admission (17.7% vs. 12.3%, *p* = 0.01). In-hospital mortality occurred in 58 (2.5%) patients [21/453 (5%) females vs. 37/1,893 (2%) males, HR = 2.28, 95% CI: 1.33–3.91, *p* = 0.003]. GBC algorithm outscored the RFC and LR models, with area under receiver operating characteristic curve (AUROC) of 0.91 with proposed working point of 83.3% sensitivity and 82.4% specificity, and area under precision recall curve (AUPRC) of 0.92. Analysis of feature importance indicated that older age, STEMI, and inflammatory markers were the most important contributing variables.

**Conclusions:**

Mortality and complications rates among females with ACS are significantly higher than in males. Machine learning algorithms for prediction of ACS outcomes among females can be used to help mitigate sex bias.

## Introduction

Although mortality associated with Acute Coronary Syndrome (ACS) has decreased in recent years thanks to improvements in prevention as well as better pharmacologic and interventional therapies, ACS and Ischemic Heart Disease (IHD) continue to be a major cause of death and disability (1–3). Recent epidemiological studies point out that the burden of this syndrome is increasing with more than 7 million people diagnosed with ACS annually worldwide (4).

Sexbiases in ACS have received increasing attention in recent decades, with numerous studies reporting significant sex-based differences in diagnosis, management, and outcomes of ACS patients (5–9). Contemporary data demonstrates that in-hospital mortality rates and the risk of recurrent cardiovascular events are higher among females with ACS when compared with males (10–12). Several factors contribute to this disparity, including increased time between symptom onset and diagnosis (13), less aggressive treatment upon diagnosis (14), and poorer in-hospital quality of care (15).

Artificial Intelligence (AI) algorithms have revolutionized healthcare by addressing a wide range of challenges, particularly in predictive tasks (16, 17). The use of AI in cardiology has been increasingly prominent, encompassing the prediction of cardiovascular disease outcomes, non-invasive diagnostics, and identification and risk assessment of life-threatening conditions (18, 19). Hence, we sought to investigate and report sex disparities in the management and outcomes of ACS patients at a tertiary care medical center using an AI-based algorithms. The aim of the current trial was to provide a proof of concept for the use of AI algorithms that are specifically designed to predict in-hospital mortality among females with ACS, and to highlight their possible role in reducing sex bias among this population of patients.

## Methods

### Study population

All patients diagnosed with ACS who were admitted to a tertiary care intensive cardiac care unit (ICCU) at Shaare Zedek Medical Center between July 2019 and July 2023 were prospectively recruited. The diagnosis of ACS was based on clinical symptoms of myocardial ischemia, with or without new ECG ischemic changes, and with or without acute elevation in high-sensitivity troponin I (hs-cTnI) concentrations, according to the ESC guidelines for ACS (20).

### Data collection

Data were anonymously documented in the ICCU by the local coordinator and prospectively submitted into an electronic case report form (eCRF). Data were checked for accuracy and out-of-range values by the coordinating unit. Demographic data, presenting symptoms, comorbid conditions, physical examination, and laboratory data were systematically recorded.

The Institutional review board approved the study based on strict maintenance of participants’ anonymity by de-identifying during database analysis. No individual consent was obtained. Moreover, the authors have no conflicts of interest to declare. No funding was applied to the study. All methods were performed in accordance with the relevant guidelines and regulations.

### Study outcomes

The primary outcome was in-hospital mortality that was recorded as an outcome for every ACS patient.

### Development and evaluation of machine-learning models

For the development of the model, only variables available at patient presentation were included, so features like culprit vessel and angiographic results were not used for model construction. The entire variables were eligible for selection in the predictive models, and no feature selection method was applied before the models training. Variables that contained missing values were not included in the analysis and are not reported here, all of the reported variables did not had missing values. The cohort of female patients with ACS was partitioned into distinct non-overlapping sets, with 70% of patients allocated to the training-validation set, and 30% assigned to the test set. The training-validation set was used for training and optimization using 5-fold cross validation. The selection of patients for each set was conducted randomly. To address the imbalance between labels, we down-sampled the training-validation set of patients who did not experience mortality during admission. All models development and parameter selection procedures were carried out exclusively on the training and validation sets, and the ultimate performance of the final model was reported based on the imbalanced test set. Our analysis includes three prediction algorithms, including gradient boosting classifier (GBC), random forest classifier (RFC), and logistic regreesion (LR). The models were optimized through a Bayesian optimization process on a set of model-specific parameters. The optimization process was carried out using a 5-fold cross-validation technique, and the best iterations were selected based on the mean area under the curve of the receiver operating characteristics curve (AUROC). We further evaluated the models based on various prediction scores including sensitivity, specificity, positive predictive value (PPV), and area under precision recall curve (AUPRC). In addition to the ROC curve, we plotted the PPV against the sensitivity (precision–recall curve). This curve enabled us to assess the clinical utility and added value of the proposed model. In order to highlight the features that influence the forecasts generated by the GBC, SHAP values (21) were calculated. SHAP values delineate the decomposition of prediction outcomes for each individual sample into the contributions attributable to distinct constituent feature values. This decomposition process is achieved through the estimation of variations between models built upon subsets of the feature space. Through the process of sample-wise averaging, SHAP values provide an assessment of the impact of each feature on the aggregate model predictions. The predictive model was developed, validated, and evaluated using Python programming language version 3.6 (Python Software Foundation).

### Statistical analysis

Continuous variables were expressed as mean ± standard deviation if normally distributed or median with interquartile range if skewed. Categorical variables were presented as frequency (%). Continuous data were compared with the Student's *t*-test and Mann–Whitney test for comparison of normally and non-normally distributed continuous variables, respectively. Categorical data were compared with the use of the chi-square test or Fisher exact test.

All statistical analyses were performed using R software version 3.4.4 (R Foundation for Statistical Computing). An association was considered statistically significant for a two-sided *P* value of less than 0.05.

## Results

### Baseline characteristics

**Figure 1:**
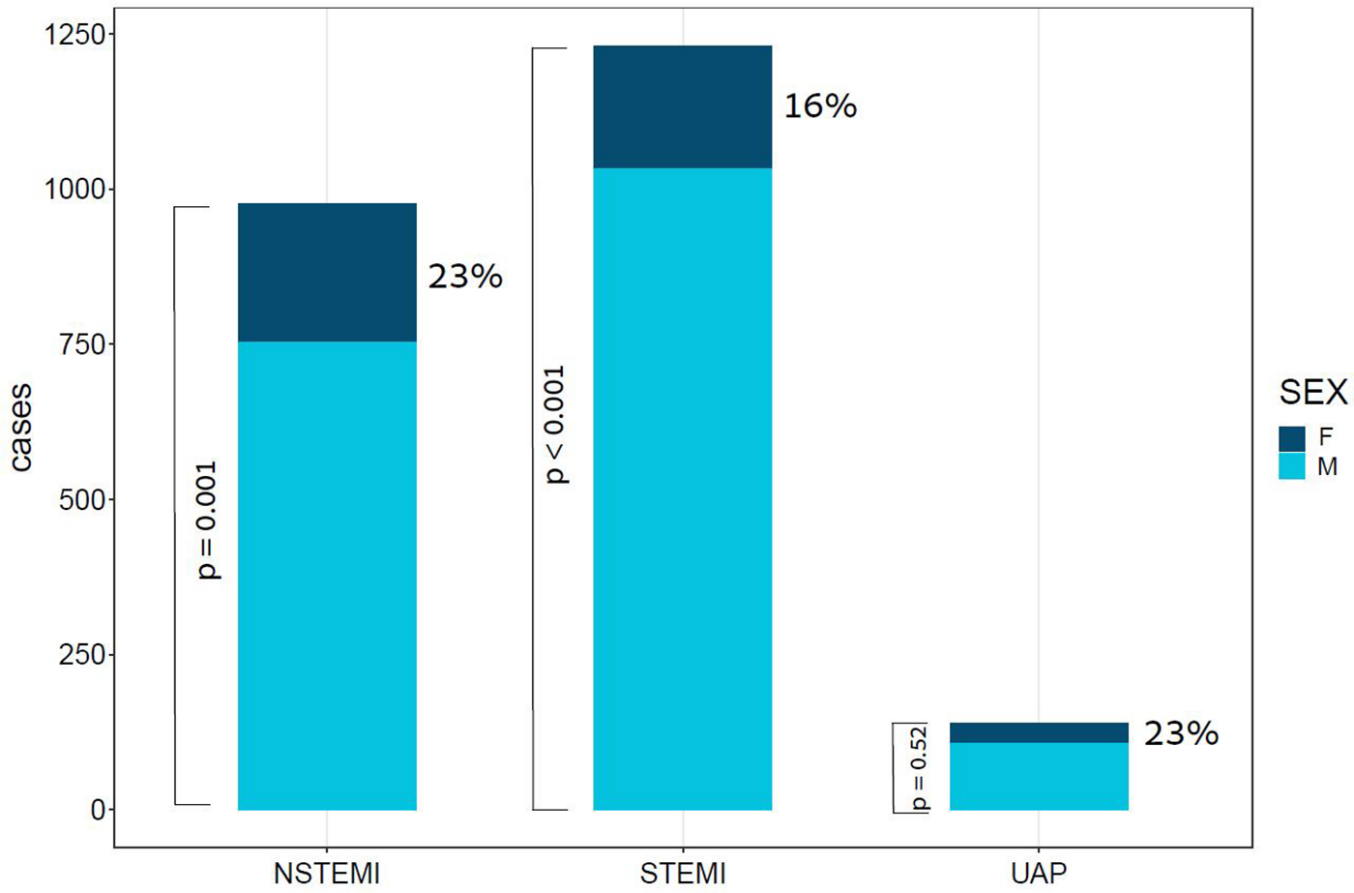
Bar plot of ACS cases by subtype and Sex. This bar plot demonstrates the relative portion of female patients in each of the subtypes of ACS. ACS, acute coronary syndrome; F, females; M, males; NSTEMI, non-ST segment elevation myocardial infraction; STEMI, ST segment elevation myocardial infraction; UAP, unstable angina pectoris; *p* value for the difference between sexes in STEMI < 0.001, in NSTEMI = 0.001, and in UAP = 0.52.

The study population comprised of 2,346 patients, with a median age of 64 [interquartile range (IQR): 56–74]. Of these patients, 453 (19.3%) were female. A total of 1,231 (52.5%) patients presented with ST-segment elevation myocardial infraction (STEMI), 976 (41.6%) presented with non-ST elevation myocardial infraction (NSTEMI), and 139 (5.9%) presented with unstable angina pectoris (UAP), as depicted in Figure 1. Stratification of baseline characteristics by sex revealed that female patients were predominantly older [median age 72 (IQR: 63–81) vs. median age 63 (IQR: 55–72), *p* < 0.001], less overweight (mean BMI 27.4 vs. 28.1, *p* = 0.033), had higher prevalence of NSTEMI (49.2% vs. 39.8%, *p* < 0.001), hypertension (68.2% vs. 57.4%, *p* < 0.001), and atrial fibrillation (8.6% vs. 4.6%, *p* = 0.003). Interestingly, females with ACS had less history of prior MI (23% vs. 34.1%, *p* < 0.001), and less family history of coronary artery disease (4.9% vs. 11.4%, *p* = 0.001). Baseline characteristics of ACS patients stratified by sex are present in Table 1.

**Table 1:** Baseline characteristics.

Variable	Females (*N* = 453)	Males (*N* = 1,893)	Overall (*N* = 2,346)	*P*-value
Age (years)	72 (63–81)	63 (55–72)	64 (56–74)	<0.001
Height (m)	1.61 ± 0.06	1.72 ± 0.07	1.70 ± 0.08	<0.001
Weight (Kg)	70.8 ± 14.7	83.7 ± 15.8	81.2 ± 16.4	<0.001
BMI (kg/m^2^)	27.4 ± 5.1	28.1 ± 5	28.0 ± 5	0.0334
Admission length (days)	2.35 ± 2	2.32 ± 1.8	2.33 ± 1.8	0.97
ACS subtype				
STEMI—no. (%)	198 (43.7%)	1,033 (54.6%)	1,231 (52.5%)	<0.001
NSTEMI—no. (%)	223 (49.2%)	753 (39.8%)	976 (41.6%)	0.00121
UAP—no. (%)	32 (7.1%)	107 (5.7%)	139 (5.9%)	0.52
HTN—no. (%)	309 (68.2%)	1,086 (57.4%)	1,395 (59.5%)	<0.001
DLP—no. (%)	260 (57.4%)	1,104 (58.3%)	1,364 (58.1%)	0.938
DM—no. (%)	188 (41.5%)	707 (37.3%)	895 (38.2%)	0.263
PAD—no. (%)	19 (4.2%)	82 (4.3%)	101 (4.3%)	0.992
CHF—no. (%)	24 (5.3%)	103 (5.4%)	127 (5.4%)	0.993
PHTN—no. (%)	9 (2.0%)	21 (1.1%)	30 (1.3%)	0.328
AFIB—no. (%)	39 (8.6%)	88 (4.6%)	127 (5.4%)	0.0037
Prior MI—no. (%)	104 (23.0%)	646 (34.1%)	750 (32.0%)	<0.001
Prior CABG—no. (%)	21 (4.6%)	116 (6.1%)	137 (5.8%)	0.477
COPD—no. (%)	31 (6.8%)	103 (5.4%)	134 (5.7%)	0.513
CKD—no. (%)	36 (7.9%)	152 (8.0%)	188 (8.0%)	0.998
RRT—no. (%)	12 (2.6%)	29 (1.5%)	41 (1.7%)	0.265
Prior CVA—no. (%)	33 (7.3%)	113 (6.0%)	146 (6.2%)	0.582
Cognitive_Decline—no. (%)	11 (2.4%)	48 (2.5%)	59 (2.5%)	0.991
Debilitated—no. (%)	8 (1.8%)	27 (1.4%)	35 (1.5%)	0.866
Malignancy—no. (%)	42 (9.3%)	96 (5.1%)	138 (5.9%)	0.00296
Anemia—no. (%)	21 (4.6%)	40 (2.1%)	61 (2.6%)	0.0101
Smoking—no. (%)	74 (16.3%)	842 (44.5%)	916 (39.0%)	<0.001
Family History of CAD—no. (%)	22 (4.9%)	216 (11.4%)	238 (10.1%)	0.001
EF (%)	49 (39–54)	49 (39–51)	49 (39–54)	0.00529
Cr (mg/dl)	0.77 (0.62–0.99)	0.9 (0.76–1.11)	0.87 (0.73–1.1)	0.207
Albumin (g/dl)	3.8 (3.5–4.1)	3.9 (3.7–4.2)	3.9 (3.6–4.2)	<0.001
Peak hs-cTnI (ng/l)	5,790 (966–37,400)	11,100 (1,690–44,100)	10,000 (1,530–42,700)	0.889
HDL (mg/dl)	29 (28–31)	29 (29–32)	29 (29–32)	0.0698
LDL (mg/dl)	97 (74–131)	99 (76–129)	99 (75–130)	1
CRP (mg/l)	0.59 (0.24–1.7)	0.49 (0.21–1.21)	0.5 (0.21–1.3)	1
HbA1c (%)	5.8 (5.5–6.6)	5.8 (5.6–6.5)	5.8 (5.6–6.5)	0.971
TSH (mIU/L)	1.76 (1.1–2.7)	1.61 (1–2.4)	1.63 (1–2.46)	0.00213
FT4 (ng/dl)	1 (0.98–1.15)	1 (0.93–1.11)	1 (0.94–1.12)	<0.001
WBC (K/ul)	9.8 (7.8–13.1)	10.3 (8.3–12.7)	10.2 (8.2–12.8)	0.622
Hb (g/dl)	12.5 (11.2–13.6)	14.3 (13–15.4)	14 (12.5–15.2)	<0.001
PLT (K/ul)	248 (202–296)	230 (191–279)	234 (194–284)	<0.001
MPV (fl)	10.9 (10.4–11.6)	10.9 (10.2–11.5)	10.9 (10.2–11.6)	0.128
IPF (%)	4.4 (3.2–5.9)	4.4 (3.1–5.8)	4.4 (3.1–5.88)	0.542
Lactate (mmol/L)	1.6 (1.4–1.9)	1.6 (1.5–2)	1.6 (1.5–2)	0.903
INR	1.07 (1–1.17)	1.09 (1–1.18)	1.09 (1–1.17)	0.963
PTT (sec)	31.1 (28.3–33)	31.1 (27.6–33.4)	31.1 (27.7–33.3)	0.0514
Fibrinogen (mg/dl)	517 (445–589)	484 (412–564)	490 (418–570)	<0.001
D-dimer (ng/ml)	813 (445–1,130)	570 (330–864)	616 (348–864)	<0.001

Values are mean ± SD, (%), or median [Interquartile range: (Q1–Q3)] for continuous variables, and number of occurences (frequency %) for categorical variables. ACS, acute coronary syndrome; AFIB, atrial fibrillation; BMI, body mass index; CABG, coronary artery bypass graft surgery; CAD, coronary artery disease; CHF, congestive heart failure; CKD, chronic kidney disease; COPD, chronic obstructive pulmonary disease; CRP, C reactive protein; CVA, cerebro-vascular accident; DLP, dyslipidemia; DM, diabetes mellitus; EF, ejection fraction; FT4, free T4; Hb, hemoglobin; HbA1c, hemoglobin A1c (glycated hemoglobin); HDL, high density lipoprotein; hs-cTnI, high sensitivity cardiac Troponin I; INR, international normalized ratio; IPF, immature platelet fraction; LDL, low density lipoprotein; MPV, mean platelet volume; PAD, peripheral artery disease; PHTN, pulmonary hypertension; PLT, platelets count; PTT, partial thromboplastin time; RRT, renal replacement therapy; TSH, thyroid stimulating hormone; WBC, white blood cells count.

### Interventions and complications during ICCU admission

Procedures that were performed during the ICCU admission course are reported in Table 2. Percutaneous coronary intervention (PCI) was performed in 1,863 (80%) patients, coronary angiography without intervention was performed in 346 (14.7%), coronary artery bypass grafting (CABG) was performed in 79 (3.4%) patients, and conservative therapy alone was assigned to only 58 (2.5%) of ACS patients. Stratification by sex demonstrated that female patients were treated more conservatively with lower urgent PCI (<2 h) rates (40.4% vs. 50.6%, *p* < 0.001). Rates of usage of more advanced therapies such as mechanical ventilation, intra-aortic balloon pump (IABP), Impella, and extra-corporeal membrane oxygenation (ECMO) were similar between sexes. The overall complication rate during admission was 13.3%, with more complications seen in females than in males (17.7% vs. 12.3%, *p* = 0.01). Females were shown to have higher rates of shock (6.4% vs. 3.1%, *p* = 0.003), significant bleeding [as indicated by Bleeding Academic Research Consortium (BARC) types 3 and 5] with a subsequent need for blood transfusion (2.9% vs. 1.2%, *p* = 0.026), and had slightly higher rates of acute renal failure (3.8% vs. 2.7%, *p* = 0.48). Patient complications during admission are presented in Table 3.

**Table 2:** Interventions during admission.

Intervention	Females (*N* = 453)	Males (*N* = 1,893)	Overall (*N* = 2,346)	*P*-value
Urgent PCI (<2 hr)—no. (%)	183 (40.4%)	957 (50.6%)	1,140 (48.6%)	<0.001
PCI—no. (%)	120 (26.5%)	603 (31.9%)	723 (30.8%)	0.0849
Coronary angiography—no. (%)	109 (24.1%)	237 (12.5%)	346 (14.7%)	<0.001
CABG—no. (%)	17 (3.8%)	62 (3.3%)	79 (3.4%)	0.974
Conservative therapy—no. (%)	24 (5.3%)	34 (1.8%)	58 (2.5%)	0.1
Pacemaker/ICD—no. (%)	8 (1.8%)	39 (2.1%)	47 (2.0%)	0.923
CPR—no. (%)	20 (4.4%)	68 (3.6%)	88 (3.8%)	0.71
Mechanical ventilation—no. (%)	30 (6.6%)	120 (6.3%)	150 (6.4%)	0.976
RRT—no. (%)	7 (1.5%)	31 (1.6%)	38 (1.6%)	0.99
IABP—no. (%)	15 (3.3%)	51 (2.7%)	66 (2.8%)	0.775
Impella—no. (%)	4 (0.9%)	15 (0.8%)	19 (0.8%)	0.981
ECMO	3 (0.7%)	9 (0.5%)	12 (0.5%)	0.882

Values are mean ± SD, (%), or median [Interquartile range: (Q1–Q3)] for continuous variables, and number of occurences (frequency %) for categorical variables. CABG, coronary artery bypass graft; CPR, cardio-pulmonary resuscitation; ECMO, extra-corporeal membrane oxygenation; IABP, intra-aortic baloon pump; ICD, implantable cardioverter defibrilator; PCI, percutaneous coronary intervention; RRT, renal replacement therapy.

**Table 3:** Complications during admission.

Complication	Females (*N* = 453)	Males (*N* = 1,893)	Overall (*N* = 2,346)	*P*-value
Any complication—no. (%)	80 (17.7%)	232 (12.3%)	312 (13.3%)	0.01
Malignant arrhythmia—no. (%)	9 (2.0%)	43 (2.3%)	52 (2.2%)	0.934
Shock^a^—no. (%)	29 (6.4%)	58 (3.1%)	87 (3.7%)	0.0033
Mechanical complication—no. (%)	4 (0.9%)	10 (0.5%)	14 (0.6%)	0.679
LV thrombus—no. (%)	1 (0.2%)	29 (1.5%)	30 (1.3%)	0.083
ARF—no. (%)	17 (3.8%)	51 (2.7%)	68 (2.9%)	0.483
Significant bleeding—no. (%)	13 (2.9%)	23 (1.2%)	36 (1.5%)	0.0364
Blood transfusion—no. (%)	13 (2.9%)	22 (1.2%)	35 (1.5%)	0.0266
Vascular complication—no. (%)	3 (0.7%)	10 (0.5%)	13 (0.6%)	0.942

Values are mean ± SD, (%), or median [Interquartile range: (Q1–Q3)] for continuous variables, and number of occurences (frequency %) for categorical variables. ARF, acute renal failure; LV, left ventricle.

^a^ any type of shock.

### In-hospital mortality and models performance

**Figure 2:**
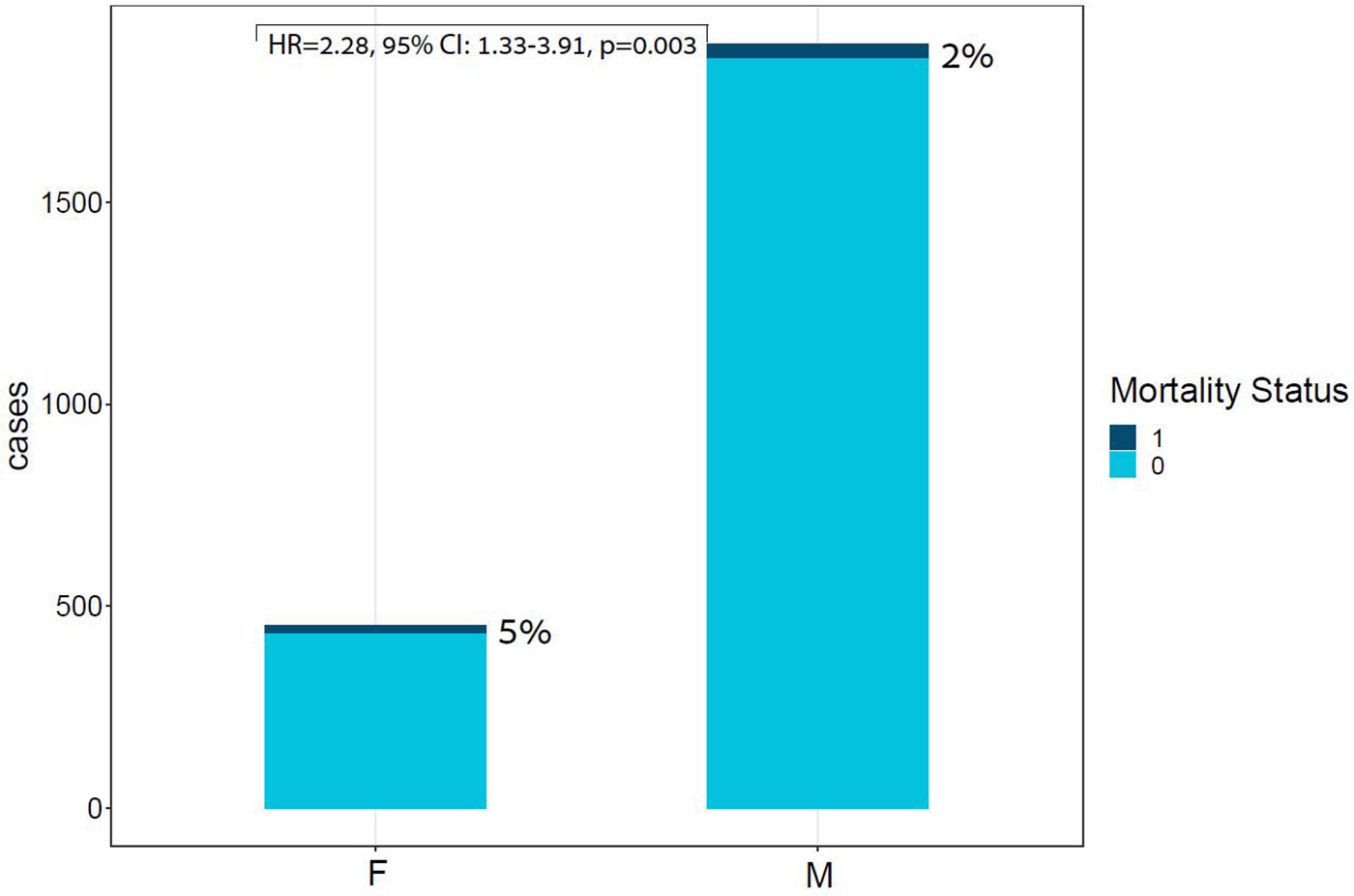
Bar plot of ACS cases by Sex and mortality Status. This bar plot demonstrates the relative portion of in-hospital mortality in each sex group, highlighting the unproportional death rates within the females subgroup. ACS, acute coronary syndrome; F, females; M, males; Unadjusted HR for sex: HR = 2.28, 95% CI: 1.33–3.91, *p* = 0.003.

**Figure 3:**
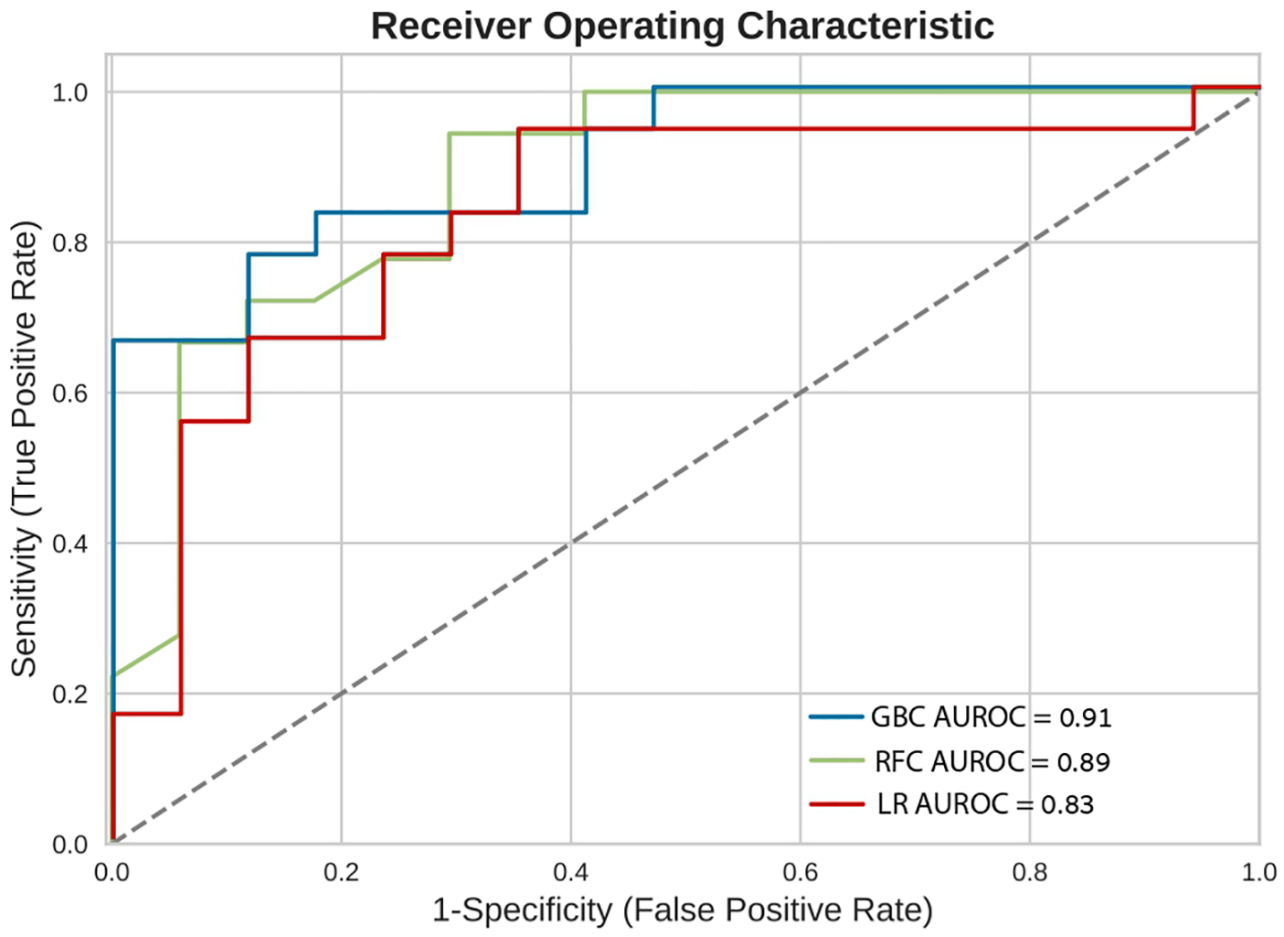
Receiver operating characteristic curves (ROC). Receiver operating characteristic (ROC) curves of the machine learning models on the selected variable set. This plot illustrates the performance of the models, including sensitivity, false positive rate, and AUROC. AUROC, area under the curve of receiver operating characterisitc curve; GBC, gradient boosting classifier; LR, logistic regression; RFC, random forest classifier.

**Figure 4:**
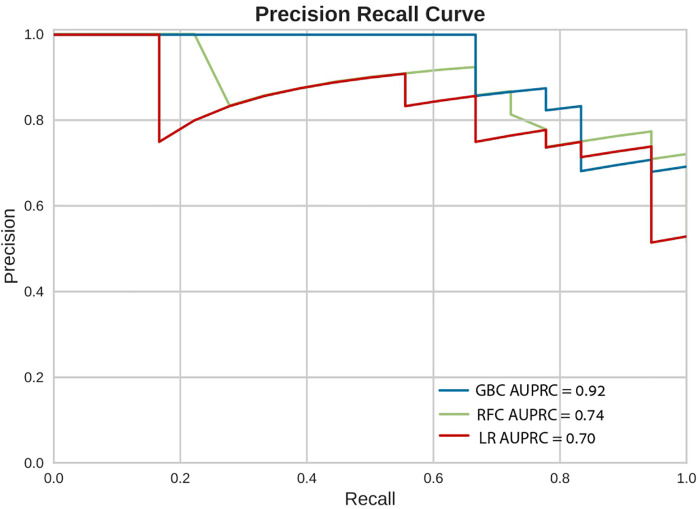
Precision recall curves (PRC). Precision Recall Curves (PRC) of the machine learning models on the selected variable set. This plot illustrates the performance of the models, including precision [positive predicted value (PPV)], recall (sensitivity), and AUPRC. AUPRC, area under the curve of precision recall curve; GBC, gradient boosting classifier; LR, logistic regression; RFC, random forest classifier.

**Figure 5:**
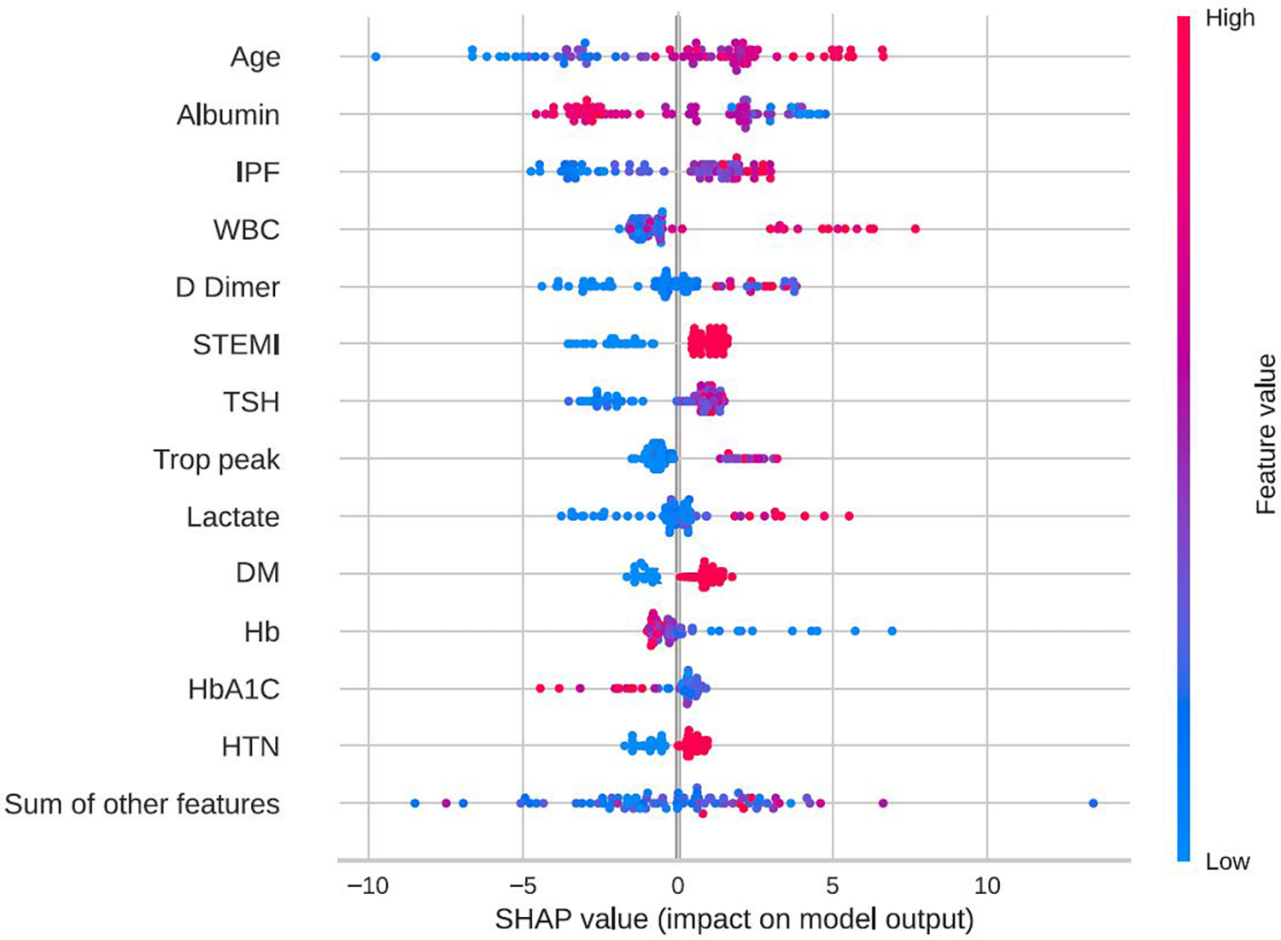
Feature importance by SHAP values. SHAP beeswarm plot of the GBC model on the selected variable set. This beeswarm plot visually depicts both the significance of variables and their effects. Every data point on the plot corresponds to a sample, with color coding indicating variable values—blue denoting low values and red signifying high values. On the X-axis, the SHAP value reflects the influence of variable values on model input, where a positive SHAP value indicates an elevated prediction probability. Moreover, greater SHAP values correlate with an increased risk of in-hospital mortality. It's essential to note that the beeswarm plot conveys the general relationship between variable values and predictions, as the color signifies relative magnitude rather than precise values. DM, diabetes mellitus; GBC, gradient boosting classifier; HbA1C, hemoglobin A1C, glycated hemoglobin; HTN, hypertension; IPF, immature platelets fraction; STEMI, ST-segment elevation myocardial infraction; Trop peak, hypersensitivity cardiac troponin I peak measure; TSH, thyroid stimulationg hormone; WBC, white blood cells count; SHAP, shapley additive explanations.

In-hospital mortality was observed in 58 (2.5%) patients. The mortality rate was found to be higher among females as compared with males (5% vs. 2%, respectively, HR = 2.28, 95% CI: 1.33–3.91, *p* = 0.003) as presented in Figure 2. Each of the three algorithms wereevaluated on the unseen test set. The GBC outperformed the other two algorithms with AUROC of 0.91 and an optimal operational threshold affording 83.3% sensitivity and 82.4% specificity. Figure 3 illustrates the ROC curves, complete with the corresponding AUROC values. Additionally, Figure 4 presents the precision-recall curves (PRC), illustrating the Positive Predictive Value (PPV) against sensitivity. The GBC outscored RFC and LR in this parameter as well, yielding an AUPRC of 0.92. The ranking of the GBC’s most influential features is summarized in Figure 5. Notably, advanced age, presentation with STEMI, evidence of diminished nutritional status (as evidenced by low serum albumin levels), and elevated inflammatory markers, were found to be strong indicators for predicting in-hospital mortality in female ACS patients. Furthermore, elevated levels of high-sensitivity cardiac troponin I (hs-cTnI), reduced serum hemoglobin levels, and heightened lactate levels were also identified as significant contributing factors.

## Discussion

Our analysis offers several important findings: First, it uses contemporary data to confirm and expand upon previous observations concerning sex disparities regarding outcomes of ACS patients. Second, this study demonstrates the potential of AI-based prediction models to mitigate these biases by providing an accurate risk estimator for sex-dependent outcomes such as in-hospital mortality. Lastly, this analysis provides an explainability layer with the use of SHAP values, which allows for the detection of important contributing variables for in-hospital mortality among females with ACS.

There are numerous sex-based differences in ACS patients. These range from basic biological features (5) (i.e., epicardial coronary artery diameter, myocardial blood flow, and estrogen-dependent endothelial mediators), as well as clinical features including risk factor profiles (7, 22), and clinical presentation and outcomes (5, 10–12). Hao et al. (6) studied sex differences in acute management, medical therapies, and in-hospital mortality in a large cohort from China. They found that females with ACS were less likely to receive evidence-based therapie than males, including reperfusion therapy. In a comprehensive review that focused on sex differences in patients with ACS in the current era (8), the researchers demonstrated higher prevalence of certain complications among females following ACS events, that included cardiogenic shock, bleeding, and post-discharge mortality. Our study findings further support the above investigations by showing that females with ACS received less aggressive treatment, most notably lower rates of urgent PCI (<2 h), and had higher rates of in-hospital complications and mortality. In our study there are several baseline characteristics that differ between males and females. The most notable difference is the older age of females compared to males, which provides a reasonable explanation for the lower rates of invasive treatment, and the higher rates of complications and in-hospital mortality between the two groups.

To the best of our knowledge, this is the first study to develop and train a machine-learning model for the prediction of in-hospital mortality exclusively for females with ACS. Prior studies have constructed models for the prediction of in-hospital mortality among all ACS patients (23, 24).

The utilization of explainability methods for the exploration of feature importance in the suggested model serves as further validation of our results. Older age and ST-segment elevation are well known risk factors for in-hospital mortality, which have previously been validated in several studies, including the most commonly used risk score for in-hospital mortality in ACS, the Global Registry of Acute Coronary Events (GRACE) (25–29). Wenzel et al. (29) developed the GRACE 3.0 score on over 400,000 patients by utilizing the GRACE parameters and applying machine learning algorithms for the prediction of in-hospital mortality, reporting and AUC of 0.91 and 0.87 for males and females with NSTEMI, respectively. Herein, we have evaluated a much smaller number of patients from a single center, but included not only NSTEMI patients but also STEMI and UAP. Moreover, we have used a variety of features in order to predict the desired outcome and not only the factors from the original GRACE score. Importantly, our study has confirmed older age and ST-segment elevation to be strong predictors of in-hospital mortality in females. Diabetes mellitus and arterial hypertension are two comorbidities that have been proposed in the Thrombolysis in Myocardial Infraction (TIMI) risk score for STEMI (27), and were also identified by our feature importance analysis as key predictors of in-hospital mortality. Interestingly, our analysis revealed the significance of other non-overlapping features linked to inflammatory markers including immature platelets fraction (IPF) (30), white blood cells (WBC) count, and D-dimer levels. These features have been associated in prior investigations with ACS pathogenesis and outcomes (31–33). Interestingly, elevated TSH and lower albumin levels were also among the most influential factors. These factors are not usually taken into account when discussing ACS prognosis but have previously reported as having prognosting implications (34, 35). Albumin and TSH are disturbed in numerous severe diseases, a situation which reflects both the poor baseline of the patients as well as an adaptation reaction for the disease state.

An important obstacle to implementing machine learning prediction algorithms in healthcare is clinician skepticism, largely because these algorithms are often not transparent. The explanatory analysis adds substantial value due to its ability to bridge this gap, making it easier for healthcare professionals to use these models and integrate them into operational healthcare systems.

### Study limitations

Our study has several limitations: (1) it was conducted in a single tertiary-care ICCU, with all its inherent limitations including referral bias. Our proposed model is based on data from our ICCU and currently lacks external validation. (2) Our analysis was based on overall in-hospital mortality rather than cardiovascular mortality. Though mortality statistics in Israel closely resemble those of the European Union, where cardiovascular death is the second most prevalent cause of death following cancer (36). (3) There may be unmeasured laboratory and clinical variables that could have been used to enhance the performance of our prediction model, including the time elapsed between symptom onset to ACS diagnosis, Killip class, NYHA functional class, and BNP. (4) While independent associations have been demonstrated, causality could not be established due to study design, hence the utilization of the proposed model in real-life clinical practice demands further prospective work.

## Conclusion

While significant efforts have been made to mitigate sex biases in the management and outcomes of patients with ACS, contemporary data indicate the persistence of such disparities. In our study, we have demonstrated the performance of an AI model in predicting in-hospital mortality among female ACS patients. Additionally, we have conducted a comprehensive feature importance analysis, highlighting the key contributing factors to this unfavorable outcome. Our study provides a proof of concept regarding the possible role of AI algorithms in reducing sex bias among females with ACS. By incorporating this model into the early stages of ACS management for female patients, we envision a potential pathway for addressing the disproportionate mortality rates experienced by this specific demographic with the aim of improving outcomes. Further prospective studies together with external validation are warranted to explore the practical application of this model in real-world healthcare settings, and to evaluate its potential role in combating sex biases in the management and outcomes of females with ACS.

## Data Availability

The datasets presented in this article are not readily available because data were generated at Shaare Zedek Medical Center. Derived data supporting the findings of this study are available from the corresponding author RL upon request. Requests to access the datasets should be directed to Ranel Loutati, ranellout@gmail.com.
